# Natural history of colonization with methicillin-resistant *Staphylococcus aureus* (MRSA) and vancomycin-resistant *Enterococcus* (VRE): a systematic review

**DOI:** 10.1186/1471-2334-14-177

**Published:** 2014-03-31

**Authors:** Erica S Shenoy, Molly L Paras, Farzad Noubary, Rochelle P Walensky, David C Hooper

**Affiliations:** 1 Division of Infectious Diseases, Infection Control Unit and Medical Practice Evaluation Center, Massachusetts General Hospital and Harvard Medical School, Boston, MA, USA; 2 Division of Infectious Diseases, Massachusetts General Hospital and Harvard Medical School, Boston, MA, USA; 3 The Institute for Clinical Research and Health Policy Studies, Tufts Medical Center, Tufts Clinical and Translational Science Institute and Tufts University, Boston, MA, USA; 4 Divisions of Infectious Diseases, Massachusetts General and Brigham and Women’s Hospital; Medical Practice Evaluation Center, Massachusetts General Hospital and Harvard Medical School, Boston, MA, USA; 5 Division of Infectious Diseases, Infection Control Unit, Massachusetts General Hospital and Harvard Medical School, Boston, MA, USA

**Keywords:** MRSA, VRE, Colonization, Carrier, Contact precautions

## Abstract

**Background:**

No published systematic reviews have assessed the natural history of colonization with methicillin-resistant *Staphylococcus aureus* (MRSA) or vancomycin-resistant *Enterococcus* (VRE). Time to clearance of colonization has important implications for patient care and infection control policy.

**Methods:**

We performed parallel searches in OVID Medline for studies that reported the time to documented clearance of MRSA and VRE colonization in the absence of treatment, published between January 1990 and July 2012.

**Results:**

For MRSA, we screened 982 articles, identified 16 eligible studies (13 observational studies and 3 randomized controlled trials), for a total of 1,804 non-duplicated subjects. For VRE, we screened 284 articles, identified 13 eligible studies (12 observational studies and 1 randomized controlled trial), for a total of 1,936 non-duplicated subjects. Studies reported varying definitions of clearance of colonization; no study reported time of initial colonization. Studies varied in the frequency of sampling, assays used for sampling, and follow-up period. The median duration of total follow-up was 38 weeks for MRSA and 25 weeks for VRE. Based on pooled analyses, the model-estimated median time to clearance was 88 weeks after documented colonization for MRSA-colonized patients and 26 weeks for VRE-colonized patients. In a secondary analysis, clearance rates for MRSA and VRE were compared by restricting the duration of follow-up for the MRSA studies to the maximum observed time point for VRE studies (43 weeks). With this restriction, the model-fitted median time to documented clearance for MRSA would occur at 41 weeks after documented colonization, demonstrating the sensitivity of the pooled estimate to length of study follow-up.

**Conclusions:**

Few available studies report the natural history of MRSA and VRE colonization. Lack of a consistent definition of clearance, uncertainty regarding the time of initial colonization, variation in frequency of sampling for persistent colonization, assays employed and variation in duration of follow-up are limitations of the existing published literature. The heterogeneity of study characteristics limits interpretation of pooled estimates of time to clearance, however, studies included in this review suggest an increase in documented clearance over time, a result which is sensitive to duration of follow-up.

## Background

Methicillin-resistant *Staphylococcus aureus* (MRSA) and vancomycin-resistant *Enterococcus* (VRE) are endemic in hospital settings and long-term care facilities (LTCF), and the prevalence of colonization is increasing [1-4]. The growing pools of colonized, and therefore isolated patients, impact patient care and burden the healthcare system [5-7]. The duration of MRSA and VRE colonization has previously been assessed in mostly small studies. Thus, pooling of these data might provide a better understanding of the natural history of colonization and the timing of clearance and thereby inform clinical care and public policy. We performed a systematic review of randomized controlled trials and observational studies that followed patients with a history of MRSA and VRE colonization and assessed study characteristics and study quality. In the absence of individual data, we pooled study-level data to calculate estimates of time to clearance of colonization.

## Methods

### Search strategy

We conducted two separate computerized searches in OVID Medline to identify relevant English-language studies including adults and published between January 1990 and July 2012. Index searches included MeSH terms for MRSA: “methicillin-resistant *Staphylococcus aureus*” or “methicillin resistance” and “colonization” or “carrier state”. For VRE, search terms included ”vancomycin resistance” and “*Enterococcus faecalis*” or “*Enterococcus*” or “*Enterococcus faecium*” and “colonization” or “carrier state”. Inclusion criteria required that a study define a population of MRSA or VRE carriers and subsequently perform at least one screening for colonization status in the absence of treatment or decolonization therapy for MRSA or VRE. Included studies provided the number of subjects cleared within a defined time period. The searches and subsequent study selection were conducted separately for MRSA and VRE.

### Study selection

Two authors (ESS and MLP) independently reviewed the abstracts of publications identified by the two searches. Publications that addressed the length of time subjects with a history of infection or colonization remained colonized or included evidence that patients were followed over time underwent full-text review for determination of inclusion and data extraction from those that met inclusion criteria. Studies with no abstract or for which it was not possible to determine if the publication contained data meeting inclusion criteria also underwent full-text review.

Colonization in both study selections was defined as having a positive culture or nucleic acid amplification assay (for MRSA or VRE) without evidence for active infection. Studies were required to report on screening from at least one anatomical site; any anatomical site for screening was permitted for inclusion. While clearance was defined by each study individually, at least one microbiological result supporting clearance was required for inclusion. For studies reporting more than one time-point of documented clearance, the latest time-point was included in the analysis. A third author (DCH) mediated any differences in interpretations regarding inclusion/exclusion.

### Data extraction

For studies meeting inclusion criteria for both searches, the following data were extracted: authors, study design, country, years of study, subject description, anatomic screening site, screening method (i.e., culture, molecular diagnostics), follow-up period (weeks), total number of subjects, number of subjects lost to follow-up, study-defined clearance, time to documented clearance for those who cleared (weeks) and the proportion of subjects with documented clearance. The role of antibiotic exposure with respect to the duration of colonization was assessed, if documented. Data were entered separately for the MRSA and VRE studies into standardized forms and verified in duplicate for consistency and accuracy. For VRE, it was noted if studies made a distinction between *E. faecalis* and *E. faecium*.

### Statistical methods

For both MRSA and VRE analyses, we collected information about the total sample size and number or percentage of subjects with documented clearance of colonization and the corresponding time after documented colonization at which the assessment of clearance was made for each included study. For studies that did not provide details on loss to follow-up, the number of clearance events was calculated as the product of the percent clearance times the total sample size. We conservatively assumed that all reported clearance events happened at the time-point reported by the study and not before.

For both MRSA and VRE analyses, we used Greenwood’s formula to estimate the standard error of each study’s reported decolonization rate [8]. This approach allowed for the generation of MRSA and VRE “champagne plots” of the reported rates of colonization clearance, where the size of each study’s data point is inversely proportional to its standard error. For the MRSA and VRE studies, we then separately fitted logistic regression models to assess the relationship between the proportion of patients with documented spontaneous clearance and the time since documented colonization. In parallel sensitivity analyses, we examined the relative influence of each study on the estimated median time to clearance. To do so, we used a jackknife method whereby each study, within the MRSA and VRE analyses independently, was sequentially removed from the dataset, and the median time to documented clearance was recalculated. Given the dependence of the analysis on the duration of follow-up, in a second set of sensitivity analyses, we restricted the MRSA studies to include only those that reported clearance on or before the maximum observed follow-up time for the included VRE studies. We then subsequently recalculated the median time to documented MRSA clearance with this restriction in place.

### Quality assessment

Cohort studies were assessed for quality using a modification of the Newcastle-Ottawa Quality Assessment Scale (NOS) developed specifically for the purposes of this review. The assessment was performed independently (ESS, MLP), with any disagreements resolved by a third author (DCH). Beyond meeting inclusion criteria for the review, the quality of randomized controlled trials included in the analyses was not assessed.

The standard NOS for cohort studies contains eight questions that assess study quality based on subject selection, comparison and outcome validation. This assessment evaluates studies on a maximum nine-point scale based on the representativeness of the exposed and non-exposed cohorts, ascertainment of exposure, comparability of the cohorts, assessment of outcome and appropriate follow-up time and loss to follow-up [9]. In the modified scale, we similarly addressed these quality measures. Selection and comparability quality was assessed by a demographic comparison between colonized and cleared subjects. Ascertainment of exposure and outcome was determined by recording whether colonization (exposure) and clearance of colonization (outcome) were determined using standard microbiological methods, and whether the study provided information about the length of time to documented clearance. Finally, the standard NOS records how long subjects were followed for outcomes assessment and loss to follow-up; in the modified NOS, we used a cutoff of three or more months of follow-up, and loss to follow-up of less than 30%. Of note, in the standard NOS, one of the key quality measures is demonstration that the outcome of interest was not present at the start of the study. For this systematic review, the outcome of interest was clearance of colonization. By definition, subjects who were not colonized at the start of the study were excluded since all subjects needed to be colonized in order to achieve the outcome of interest.

## Results

### MRSA

#### MRSA study identification

**Figure 1:**
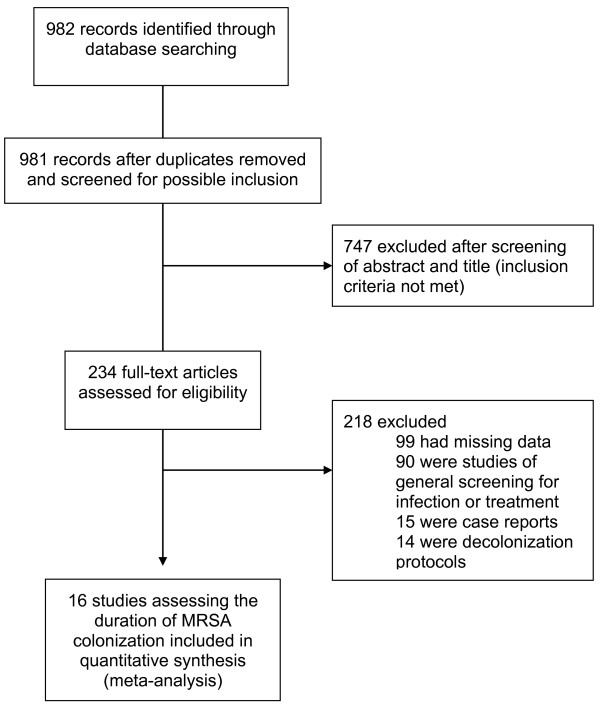
Study selection process (MRSA).

The MRSA search criteria identified 981 non-duplicate publications. Full-text review was completed for 234 publications, and each was reviewed in detail for final determination of inclusion and for data extraction (Figure 1). This procedure resulted in a total of 16 studies with 1,804 non-duplicated subjects included in the review, and 13 cohort studies included in the quality assessment (Figure 1) [10].

#### MRSA study characteristics

The 16 studies were diverse in design, geographic site, enrollment period, anatomic screening site, and patient location (Table 1) [11-26]. The median duration of total follow-up was 38 weeks.

**Table 1:** Studies documenting duration of MRSA colonization meeting inclusion criteria

**Author year [ref]**	**Study ID**	**Design**	**Country**	**Year of study**	**Subject description**	**Screening site**	**Screening method**	**Latest documented follow up (weeks)**	**N**	**Lost to follow up (N)**	**Clearance defined**	**Weeks to documented clearance**	**% cleared**
Sanford 1994 [11]	A	Retrospective cohort	USA	1989-1991	Hospitalized patients	Nares, axillae, perineum/ groin, wound, sputum, tracheal aspirate	CX; T	240	36	*	2 negative CX	172	50%†
Mulhausen 1996 [12]	B	Prospective cohort	USA	1991-2	Residents at LTCFs	Nares	CX	52	47	34	1 negative CX on two separate samplings	52	13%
O’Sullivan 2000 [13]	C	Prospective cohort	Ireland	1994-5	Residents at LTCFs	Nares, throat, hairline, axillae, groin, perineum, skin lesions	CX; T	39	65	14	1 negative CX	26	49%
Scanvic 2001 [14]	D	Prospective cohort	France	1998	Hospitalized patients	Nares, skin, axillae, groin	CX; T	36 (a)	78	*	4 negative CX obtained from 2 sites	37	50%†
Ellis 2004 [15]	E	Prospective cohort	USA	2003	US Army personnel	Nares	CX; T	13	24	*	1 negative CX	9	67%
Cretnik 2005 [16]	F	Prospective cohort	Slovenia	2001-2	Residents and HCW at LTCFs	Nares, skin lesions, axillae, groin	CX; T	13	12	2	2 negative CX from 2 sites on 3 separate samplings	13	33%
Vriens 2005 [17]	G	Prospective cohort	Netherlands	1991-2001	Hospitalized patients	Nares, throat, perineum, wounds, skin lesions, urine and sputum	CX; PCR	104	57	21	All negative CX from up to 7 sites on 3 separate samplings	104	46%
Marschall 2006 [18]	H	Retrospective cohort	Switzerland	2000-3	Hospitalized patients	Nares, groin, skin lesions, tracheal secretions, urine	CX; T	231	80	*	All negative CX from up to 6 sites on 2 separate samplings	85	50%†
Ellis 2007 [19]	I	RCT	USA	2005	US Army personnel	Nares	CX; T	16	66	1	1 negative CX	16	64%
Simor 2007 [20]	J	RCT	Canada	2000-3	Hospitalized patients	Nares, perineum, skin lesions, catheter sites	CX; T	34	35	26	1 negative CX on 2 separate samplings	13	23%
Wendt 2007 [21]	K	RCT	Germany	2001-4	Hospitalized patients and residents at LTCFs	Nares, throat, groin, perineum, skin defects, any previously colonized site	CX; T	13	58	3	All negative CX at up to 7 sites on 5 separate samplings	4	12%
Lucet 2009 [22]	L	Prospective cohort	France	2003-4	Hospitalized patients discharged to home care	Nares, chronic skin lesions	CX	52	148	44	1 negative CX on 2 separate samplings	52	51%
Robicsek 2009 [23]	M	Retrospective cohort	USA	2006-7	Hospitalized patients	Nares	PCR	208	824	*	1 negative PCR	208	79%
Lautenbach 2010 [24]	N	Prospective cohort	USA	2008	Ambulatory patients and household contacts	Nares, axilla, throat; groin and perineum	CX; PCR; T	14	11	0	All negative cultures from up to 5 sites on 6 separate collections	14	73%
Manzur 2010 [25]	O	Prospective cohort	Spain	2005-7	Residents at LTCFs	Nares, decubitus ulcers	CX; T	77	231	104	1 negative CX on 2 separate collections	77	27%
Van Velzen 2011 [26]	P	Retrospective cohort	Scotland	2010	Hospitalized patients	Nares, perineum, axillae, throat, wounds and devices	CX; PCR; T	4	32	0	All negative cultures from up to 6 sites on 2 separate occasions	1	25%

(a) Follow up at least 13 weeks since hospitalization; median clearance at 37 weeks reported; mean time since hospital discharge 36 weeks.

*Not documented; † Kaplan Meier estimates which do not provide information on those lost to follow up.

HCW: health care worker; LTCF: long term care facility; MRSA: methicillin resistant *Staphylococcus aureus*; N: number; CX: culture; T: typing of strain performed; PCR: polymerase chain reaction; RCT: randomized controlled trial.

For studies that included hospitalized patients, data were more frequently not provided about the subjects’ residence prior to admission (i.e., home versus facility). Fourteen of 16 studies sampled subjects at least three months after documented colonization. For loss to follow-up, seven studies had less than 30%, four had more than 30%, and five provided no information.

#### MRSA clearance rates

**Figure 2:**
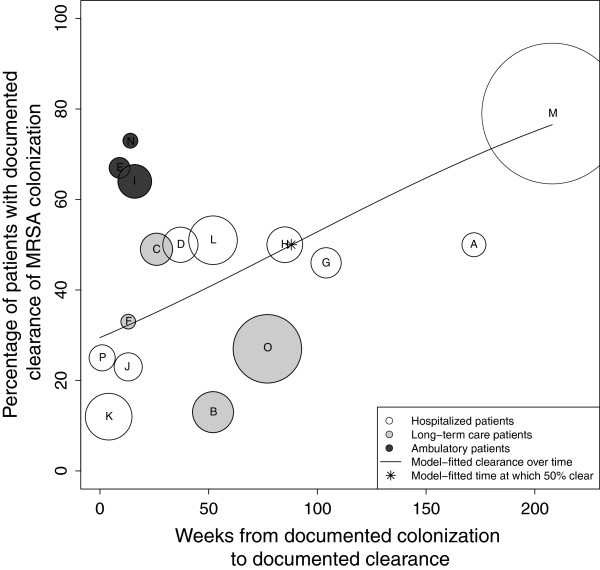
**Analysis of median time to documented clearance of MRSA colonization.** The X-axis represents time (in weeks) from documented colonization and the Y-axis represents the proportion of patients with documented clearance of colonization. This proportion included the initial subject population minus those lost to follow-up. Each circle represents a single study (A-P). The size of the circle is inversely proportional to its standard error. The line represents the model-fitted clearance over time, with 50% of subjects clearing MRSA at 88 weeks from time of documented colonization (*).

Reported clearance rates ranged from 12% [21] to 79% [23]. The time to observed clearance ranged from one [26] to 208 weeks [23]. A plot of the percentage of subjects documented to have cleared MRSA colonization over time demonstrates a trend toward the majority of subjects clearing colonization over the follow-up periods reported (Figure 2). Using logistic regression, we found that 50% of patients cleared colonization at 88 weeks after initial documentation of colonization. At one, two, three and four years after initial determination of MRSA colonization, the model-estimated proportions of subjects with documented clearance of colonization were 41, 54, 66, and 77%, respectively. Comparing the patient populations represented, long-term care, hospitalized, and ambulatory, at 26 weeks after initial documentation of colonization, the model-fitted percentages of those clearing were significantly different at 22, 36, and 68%, respectively (P < 0.0001).

The majority of studies did not provide enough information to determine if the cohorts of colonized or cleared subjects were similar. However, three studies [14,22,25] did provide these data, and in general the demographic characteristics of the subjects in the colonized and cleared groups from each cohort were similar, with some notable exceptions. Scanvic reported residence at another healthcare institution and break in the skin to be significantly associated with persistent carriage (32% vs 11% and 67.7% vs. 28%, respectively). Lucet found assistance with activities of daily living (ADLs) to be significantly different between the groups (57.5% vs. 49.3%, respectively). Manzur reported the presence of decubitus ulcers to be a risk factor for persistent colonization (27.5% vs. 13.7%, respectively).

The jackknife sensitivity analysis revealed that, for all but two studies ([25], Study “O” and [23], Study “M”), the estimated median time to documented clearance would change only minimally if each study was excluded. The exclusion of Manzur [25] resulted in a model-fitted median time to clearance of 68 weeks. Separately, the exclusion of Robicsek [23] resulted in a model-fitted median clearance time >172 weeks after documented colonization.

The impact of antibiotic exposure on persistence of colonization was either not reported or found to have no significant association with duration of colonization for the majority of studies. Only one study reported that antibiotic exposure in colonized patients was significantly associated with persistence of colonization [18].

#### MRSA study quality

The 13 cohort studies were assessed using the modified NOS. Of these, only Lucet [22] met all quality criteria based on the modified NOS. The remainder of the studies were missing quality criteria in either comparability, appropriate time to follow-up or loss of follow-up; overall, these studies were of moderate quality, with the mean score of 4.8/6 (range 3–6). As the NOS results lacked variation and were applicable only to cohort studies, they were not used as weights in the pooled analyses.

### VRE

#### VRE study identification

**Figure 3:**
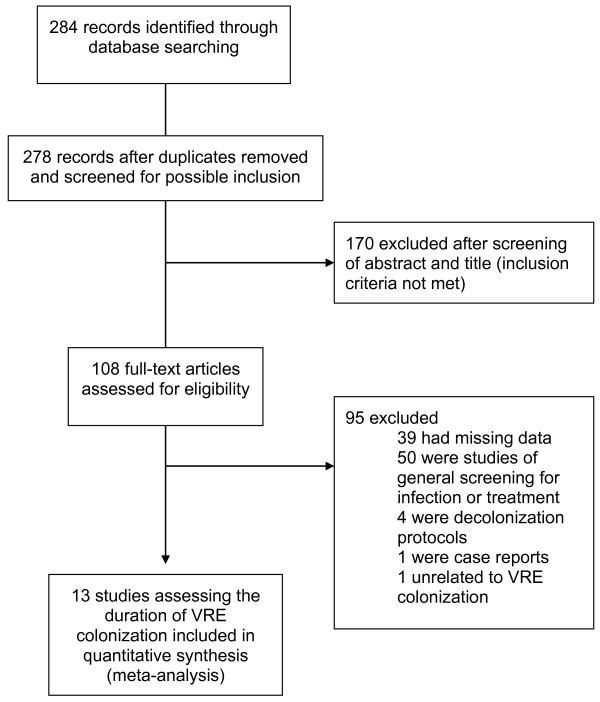
Study selection process (VRE).

The search criteria identified 278 non-duplicate screened publications. Full-text review was completed for 108 publications, all of which were reviewed in detail for final determination of inclusion and data extraction. This procedure resulted in 13 studies included in the review, and 12 cohort studies included in the quality assessment (Figure 3) [10].

#### VRE study characteristics

The 13 included studies, including a total of 1,936 non-duplicated patients varied in design, geographic site, enrollment period, and anatomic screening site; the vast majority included hospitalized patients (Table 2) [27-39]. The median duration of total follow-up was 25 weeks. Seven of the 13 studies reported on *E. faecium* alone, while six of 13 made no distinction between *E. faecalis* and *E. faecium*.

**Table 2:** Studies documenting duration of VRE colonization meeting inclusion criteria

**Author year [ref]**	**VRE**	**Study ID**	**Design**	**Country**	**Year of study**	**Subject description**	**Screening site**	**Screening method**	**Latest documented follow up (weeks)**	**N**	**Lost to follow up (N)**	**Clearance defined**	**Weeks to documented clearance**	**% cleared**
Montecalvo 1995 [27]	VREF	A	Prospective cohort	USA	1993-4	Hospitalized patients	Perianal	CX; T	18	86	50	1 negative CX on admission and weekly negative culture during admission	18	2%
Brennen 1998 [28]	VREF	B	Prospective cohort	USA	1993-4	Residents in LTCFs	Rectal	CX	25	36	*	2 negative CX on 2 separate samplings	10	50%†
Goetz 1998 [29]	VREF	C	Prospective cohort	USA	1994-6	Hospitalized patients	Rectal or stool	CX	*	210	61	1 negative CX on 2 separate samplings	14	40%†
Bhorade 1999 [30]	ND	D	Prospective cohort	USA	1996-8	Hospitalized patients	Rectal or stool	CX	2	10	6	1 negative CX on 5 separate samplings	N/A	0%
Weinstein 1999 [31]	VREF	E	Prospective cohort	Canada	1995	Hospitalized patients	Rectal	CX	25	24	0	1 negative CX on at least 3 separate samplings	25	38%
D’Agata 2001 [32]	ND	F	Prospective cohort	USA	1998	Hospitalized patients	Rectal	CX	3	13	6	1 negative culture on at least 2 separate samplings	1	8%
Wong 2001 [33]	ND	G	RCT	USA	*	Hospitalized patients and residents of LTCFs	Rectal	CX	3	24	4	1 negative CX on 3 separate samplings	3	21%
Byers 2002 [34]	ND	H	Retrospective cohort	USA	1994-6	Hospitalized patients	Rectal	CX; T	86	116	0	1 negative CX on 3 separate samplings	22	64%
Hachem and Raad 2002 [35]	VREF	I	Prospective cohort	USA	1997	Hospitalized patients	Stool	CX	13	28	0	1 negative CX on at least 2 separate samplings	13	4%
Mascini 2003 [36]	VREF	J	Prospective cohort	Netherlands	2000	Hospitalized patients	Rectal	CX; PCR;T	26	11 (a)	*	3 negative CX on at least 3 separate samplings	6	50%†
Huang 2007 [37]	ND	K1	Retrospective cohort	USA	2002-4	Hospitalized patients	Rectal	CX; PCR (b)	52	394 (c)	*	1 negative CX	9	24%
Huang 2007 [37]	ND	K	Retrospective cohort	USA	2002-4	Hospitalized patients	Rectal	CX; PCR (b)	52	126 (d)	*	1 negative CX	43	84%
Park 2011 [38]	ND	L1	Retrospective cohort	South Korea	2003-10	Hospitalized patients on chronic HD	Rectal	CX	39	89	20	1 negative CX on 3 separate samplings	16	10%
Park 2011 [38]	ND	L	Retrospective cohort	South Korea	2003-10	Hospitalized patients on non-chronic HD	Rectal	CX	35	723	339	1 negative CX on 3 separate samplings	9	12%
Yoon 2011 [39]	VREF	M	Retrospective cohort	South Korea	2006-9	Hospitalized patients	Rectal	CX	19	58	*	1 negative CX on 3 separate samplings	19	28%

(a) Only patients with non-epidemic strain were included in analysis. (b) One of four participating sites used both culture and PCR; the others used culture only. (c) Subset of patients admitted ≤ 60 days from last known positive culture. (d) Subset of patients admitted > 300 days from last known positive culture.

*Not documented; † Kaplan Meier estimates which do not provide information on those lost to follow up.

HCW: health care worker; HD: Hemodialysis; LTCF: long term care facility; VRE: vancomycin resistant enterococcus; VREF: *E. faecium*; ND: no distinction made between *E. faecalis* and *E faecium*; N: number; CX: culture; T; strain typing performed; PCR: polymerase chain reaction; RCT: randomized controlled trial.

#### VRE clearance rates

**Figure 4:**
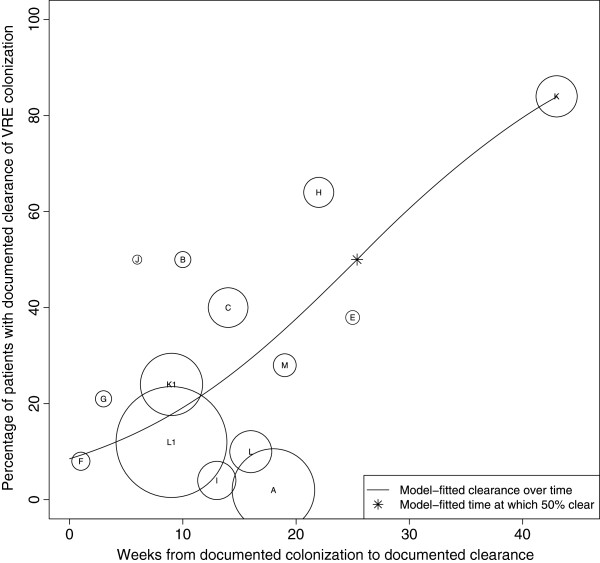
**Analysis of median time to documented clearance of VRE colonization.** The X-axis represents time (in weeks) from documented colonization and the Y-axis represents the proportion of patients with documented clearance of colonization. This proportion included the initial subject population minus those lost to follow-up. Each circle represents a single study (A-M). The size of the circle is inversely proportional to its standard error. The line represents the model-fitted clearance over time, with 50% of subjects clearing VRE at 26 weeks from time of documented colonization (*).

Reported time to clearance ranged from one [32] to 43 weeks [37], with documented clearance rates of zero [30] to 84% [37]. A plot of the percentage of subjects documented to have cleared VRE colonization over time demonstrates a trend toward the majority of subjects clearing colonization during the period of observation (Figure 4). Using logistic regression, we found that 50% of subjects cleared colonization at 25 weeks after initial documentation of colonization. At 10, 20, 30 and 40 weeks after initial determination of VRE colonization, the model estimated that 19, 38, 61, and 80% of subjects had documented clearance of colonization, respectively. Since the vast majority of subjects were hospitalized patients, we were unable to assess the effect of patient population type (inpatient versus LTCF resident) on time to documented clearance.

Only two of the studies included, Park [38] and Yoon [39] provided sufficient demographic data to formally evaluate variables associated with either persistence or clearance of colonization. Park [38] found three variables, age (odds ratio [OR]: 0.99; P = 0.05), duration of glycopeptide use prior to VRE positivity (OR: 2.16; P = 0.003), and length of hospital stay (OR 1.01; P = 0.001) associated with subjects having three consecutive negative rectal cultures. Mean duration of glycopetide use was reported with respect to hemodialysis; patients on chronic HD were observed to be exposed to 12.7 days while patients on non-chronic HD were observed to be exposed to 4.5 days (P = 0.001). Yoon [39] compared subjects who cleared colonization early (< three weeks) to those who cleared later (≥ five weeks) and found that they differed on several characteristics. The early group was more likely to be younger (P = 0.01) and to have a shorter length of stay in an ICU (P = 0.03). This group was also less likely to have had prior exposure to medical devices, including central venous catheters and endotracheal intubation (P = 0.04 and P = 0.04, respectively), and less likely to have received selected antibiotics after colonization, including carbapenems (P = 0.01), vancomycin (P < 0.001), or fluoroquinolones (P = 0.04). In multivariable logistic regression analysis, they found that vancomycin use after VREF colonization was significantly associated with prolonged carriage (OR 4.1; P = 0.02).

Unlike for the MRSA analysis, the VRE jackknife sensitivity analysis revealed that the estimated median time to documented clearance did not appear substantially different with the exclusion of any one study.

Antibiotic exposure was reported to be significantly and positively associated with longer duration of carriage in several of the studies [28,34,39], however others found no such association [33,36,38]. The remaining studies did not report on the relationship.

#### VRE study quality

The 12 cohort studies were assessed using the modified NOS. No study fulfilled all quality criteria for the modified NOS; the majority were of moderate quality with the mean score of 4.6/6 (range 3–5). As the NOS results lacked variation and were applicable only to cohort studies, they were not used as weights in the pooled analyses.

### Comparison of MRSA and VRE pooled clearance rates

**Figure 5:**
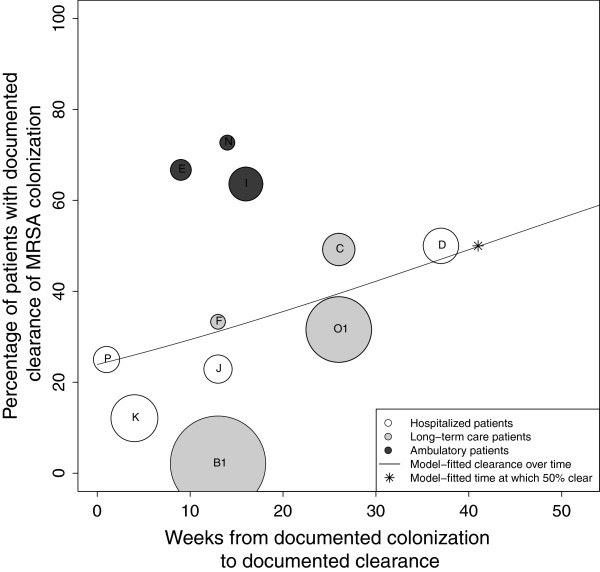
**Analysis of median time to documented clearance of MRSA colonization, restricted follow-up period.** The X-axis represents time (in weeks) from documented colonization and the Y-axis represents the proportion of patients with documented clearance of colonization. This proportion included the initial subject population minus those lost to follow-up. Each circle represents a single study as in Figure 2, excluding studies A, G and H). The size of the circle is inversely proportional to its standard error. The line represents the model-fitted clearance over time, with 50% of subjects clearing MRSA at 41 weeks from time of documented colonization (*).

Clearance rates for MRSA and VRE were compared by restricting the time to follow-up for the MRSA studies to the maximum observed time point for VRE studies (43 weeks). Figure 5 shows that with this restriction, which results in the exclusion of five studies (A, G, H, L, M) and the inclusion of earlier-reported time intervals from two studies (Manzur, “O1” and Mulhausen, “B1”), the model-fitted median time to documented clearance would occur at 41 weeks.

## Discussion

We reviewed the published literature on the natural history of MRSA and VRE colonization. There is substantial heterogeneity among the included studies. This heterogeneity both identifies a clear need for further investigation and tempers our interpretation of the pooled estimates of time to clearance of colonization. Based on the studies meeting our inclusion criteria, our systematic review demonstrates that persistent colonization decreases over time, with clearance of colonization in half of patients at 88 weeks for MRSA and 26 weeks for VRE.

While the weight of the existing data, and clinical experience, suggest that clearance of MRSA and VRE colonization increases over time, precision around the time to clearance is not possible due to the major limitations of the studies in this domain. These limitations not only hinder our interpretation of the pooled findings, but also highlight the need for additional studies. Lack of a consistent definition of clearance, uncertainty regarding the time of initial colonization, variation in frequency of sampling for persistent colonization, and variation in duration of follow-up and loss to follow-up all impose substantial constraints on our interpretation of the median time to clearance.

Because subjects are not screened prospectively and continuously, the initial date of colonization is assumed to be the time MRSA or VRE were discovered and documented (based on either a clinical infection or a surveillance screening). There is almost certainly variation in the lag time between actual colonization and the identification of a subject as being colonized. Under these inevitable circumstances, calculations of duration of colonization may underestimate the true duration of colonization and the pattern of clearance. On the other hand, if the time interval between initial documentation of colonization and re-screening is prolonged, duration of colonization may also be overestimated. A striking observation from the combined reviews is the relatively short time to clearance for VRE, as compared to MRSA. The longer median time to clearance estimated for MRSA is in part a reflection of the length of follow-up, as evidenced by the reduction from 88 weeks to 41 weeks observed when follow-up was restricted to the same duration as the VRE studies.

We did not impose a universal definition of clearance as a prerequisite for inclusion in our review, and across studies, the definition of clearance varied in part due to the absence of guidelines that define clearance of colonization [40]. The diversity of results that were considered evidence of clearance across the studies is a reflection of the lack of consensus on this point, and limits our interpretation of the pooled estimates. The frequency of re-sampling and duration of follow up varied as well. These factors would be expected to affect reported time to clearance and thus add reasons to be cautious in the interpretation of the systematic review. Several of the MRSA studies [11,14,18,22,23,25] showed an initial brisk decline in colonization followed by a stabilization of the pool of colonized (in those studies following patients for extended periods), supporting the general consensus that there are likely sub-groups among colonized patients including those who are transiently, intermittently or persistently colonized. While some of the studies did make such distinctions, again, the definition of each carrier-state varied.

Beyond the concepts of transient, intermittent or persistent colonization, isolates identified in the screening studies may represent an initial colonizing strain or a second (or third) isolate. Some studies performed additional analysis to identify strain types. In the absence of strain-typing, it is not possible to conclude that a patient who remains persistently colonized is in fact colonized with the endogenous strain, or intermittently colonized with different strains. From the perspective of infection control implementation, such distinctions may not be meaningful in terms of the practical implementation of CP measures, and those cases in which cohorting is permitted [40,41]. Given currently available assays, documented clearance of colonization may in fact represent a level of colonization below the limits of detection (with the same strain or different strain).

A conceptual limitation of our review relates to colonization dynamics and specifically the clearance of the colonizing strain or re-colonization with a new strain, which may be particularly relevant in the setting of selective antibiotic pressure. In the VRE analysis, one clinical variable, prior antibiotic use, was associated with a trend toward early clearance of colonization, supporting the observation that concurrent antibiotic therapy affects the sensitivity of surveillance cultures for VRE [42,43]. The issue of test sensitivity is particularly relevant in the setting of selective antibiotic pressure, as has been demonstrated in the case of VRE [44-47]. A detailed analysis of the impact of antibiotics on colonization dynamics was not directly within the scope of the study, however, is an important area for further investigation, especially with respect to VRE. In the VRE studies included in this review, the lack of universal definitions of clearance and carriage-type again limit our interpretation. Finally, the years included span close to 20 years for both MRSA and VRE, during which changes in epidemiology, screening practices, and technology have taken place, raises challenges for comparison across studies and interpretation of the pooled clearance estimates.

Additional limitations are second-order compared to the fundamental deficiencies described above. Most studies provided insufficient details regarding the demographic characteristics of subjects who cleared versus those who remained persistently colonized. However, one demographic variable for MRSA-colonized patients, ambulatory patient status versus inpatient or LTCF patient status, was associated with a trend toward early clearance of colonization in three studies. It is possible that the persistence of colonization in hospitalized and LTCF patients may be the result of re-colonization. Based on our quality assessment of cohort studies, the majority were of moderate quality.

Our analysis was also limited by the use of aggregate data rather than patient-level data, which would have permitted a survival analysis approach. Most studies screened using microbiological methods that relied on standard culture techniques. Although molecular assays are more costly on a per-test basis, their greater sensitivity may allow reduction in repetitive testing and more effective implementation [48]. Additional research employing molecular methods in studies of the natural history of colonization is needed. Even with the more widespread use of molecular assays however, it is still possible that colonized patients may fail to be identified, if the level of colonization is below the limit of the sampling methods. In terms of practical implications of false negative screens, patients with low levels of colonization may pose a lower transmission risk. Finally, we are not able to address the risk of publication bias in the inclusion of studies.

## Conclusions

Our study is the first systematic review to address the topic of time to clearance of MRSA and VRE colonization. Our review highlights a substantial degree of heterogeneity across the studies, beyond those common in such analyses. The fundamental differences across studies including definition of clearance of colonization, frequency of sampling, assays implemented and duration of follow up, highlight the gaps in the available data and caution the interpretation of estimates of clearance derived from pooling the studies included. Despite the strengths and weaknesses of the existing literature and the methodological challenges of interpreting pooled results across a heterogeneous group of studies, the data suggest a decline in colonization over time. While there is uncertainty about the complete duration of colonization, the types of data in the included studies are those generally available in clinical settings and thus can set a time frame for clearance in most patients. The analysis presented brings to the fore the inconsistencies with infection control policies that assume colonization is life-long.

The duration of colonization has important implications for patient care, infection control policy, and resource utilization. Once a patient is known to have a clinical infection or to be colonized with MRSA or VRE, he or she is usually placed on contact precautions (CP) based on guidelines from the Centers for Disease Control and Prevention (CDC) [41]. Many institutions document such patients as MRSA or VRE carriers, so that on readmission they are again placed on CP. CDC has not provided guidance on when or under what testing circumstances CP may be discontinued, resulting in nationwide variation in policies and procedures regarding the duration of CP for MRSA and VRE [49]. If patients labeled as carriers have in fact cleared colonization, they are likely exposed to the various adverse consequences of CP with additional costs [5,6,50-56].

Further research is needed to address the lag time from initial colonization to documented colonization and to address the issue of sampling bias (both frequency and duration of follow-up). Prospective studies of the natural history of colonization, based on consensus definitions of colonization and clearance, are needed. Such studies will be critical for informing screening policies for identifying those patients no longer colonized with MRSA or VRE and to support guidelines on duration of contact precautions.

## Abbreviations

MRSA: Methicillin-resistant *Staphylococcus aureus*; VRE: Vancomycin-resistant Enterococcus; LTCF: Long-term care facility; CP: Contact precautions.

## Competing interests

The authors declare that they have no competing interests.

## Authors’ contributions

ESS and DCH conceived of the study. ESS, MLP, FN, RPW and DCH participated in the design of the study. ESS, MLP and FN performed the analysis. ESS, MLP, FN, RPW and DCH analyzed the data. ESS and MLP wrote the first draft of the manuscript and ESS, MLP, FN, RPW and DCH contributed to the writing of the manuscript and approved the final manuscript.

## Pre-publication history

The pre-publication history for this paper can be accessed here:

http://www.biomedcentral.com/1471-2334/14/177/prepub
